# High-mobility group box 1 protein, histone H3 and histone H4 are not associated with peripheral hypoperfusion in sepsis: a retrospective cohort study

**DOI:** 10.31744/einstein_journal/2026AO1615

**Published:** 2026-04-30

**Authors:** Bruna Cassia Dal Vesco, Ana Carolina de Miranda, Fernanda do Carmo De Stefani, Luis Gustavo Morello, Dalila Luciola Zanette, Luiz Eduardo Nunes Ferreira, Igor Alexandre Cortês de Menezes

**Affiliations:** 1 Universidade Federal do Paraná Hospital de Clínicas Department of Internal Medicine Curitiba PR Brazil Department of Internal Medicine, Hospital de Clínicas, Universidade Federal do Paraná, Curitiba, PR, Brazil.; 2 Fundação Oswaldo Cruz Instituto Carlos Chagas Laboratory of Applied Science and Technology in Health Curitiba PR Brazil Laboratory of Applied Science and Technology in Health, Instituto Carlos Chagas, Fundação Oswaldo Cruz (FIOCRUZ), Curitiba, PR, Brazil.; 3 Universidade Guarulhos Laboratory of Inflammation and Immunology Guarulhos SP Brazil Laboratory of Inflammation and Immunology, Universidade Guarulhos, Guarulhos, SP, Brazil.

**Keywords:** Sepsis, Alarmins, HMGB1 protein, Histones, Perfusion index, Microcirculation

## Abstract

**Objective:**

The causal mechanisms of peripheral hypoperfusion in sepsis have not yet been fully elucidated. Therefore, this study aimed to investigate whether peripheral hypoperfusion in septic patients is associated with serum levels of damage-associated molecular patterns.

**Methods:**

A retrospective cohort study was conducted to compare serum levels of damage-associated molecular patterns - histone H3, histone H4, and high-mobility group box-1 protein (HMGB1) - with markers of tissue hypoperfusion - the peripheral perfusion index and the capillary refill time, using a single measurement obtained within the first 24 hours after sepsis diagnosis in critically ill patients from two Brazilian hospitals.

**Results:**

Eighty patients were included in the study. Approximately half of the patients showed persistent peripheral hypoperfusion as determined by at least one of the techniques used. Surviving patients had better capillary refill time [2 (2-4) *versus* 4 (2-5), p=0.01] and perfusion index [1.9 (0.98-4.95) *versus* 0.54 (0.32-0.87) p<0.0001] compared with non-survivors. None of the evaluated damage-associated molecular patterns distinguished between survivors and non-survivors. In addition, damage-associated molecular patterns were not associated with perfusion index (versus HMGB1, p=0.13; *versus* H3, p=0.51 and *versus* H4, p=0.25) or capillary refill time (*versus* HMGB1, p=0.29; *versus* H3, p=0.60 and *versus* H4, p=0.92).

**Conclusion:**

In septic patients evaluated in this retrospective cohort study, no statistically significant association was found between the levels of histones H3, H4, and HMGB1 protein, tissue hypoperfusion, and mortality.

## In Brief

Damage-associated molecular patterns mediate the immuneinflammatory response in sepsis, a process closely related to multiple organ dysfunction. Peripheral hypoperfusion has also been linked to increased mortality in septic patients. Therefore, this retrospective cohort study evaluated the relation between histone H3, histone H4, and high-mobility group box 1 protein with peripheral perfusion in sepsis.

## Highlights

■ This study aimed to evaluate the association between damage-associated molecular patterns and peripheral hypoperfusion in sepsis.
■ Surviving patients showed better peripheral perfusion than non-survivors.
■ No significant association was observed between mortality and serum levels of the evaluated damage-associated molecular patterns.
■ Damage-associated molecular patterns levels did not differ between patients with reduced or normal peripheral perfusion.

## Graphical abstract

**Figure:**
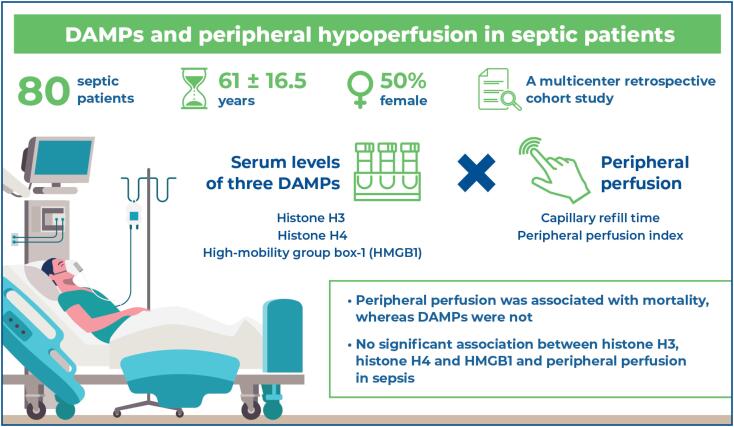

## INTRODUCTION

Sepsis remains the leading cause of death among critically ill patients.^(1,2)^ For this reason, understanding the pathophysiological mechanisms behind the syndrome is of major importance to translate this knowledge into concrete results at the bedside.^(3,4)^

A condition related to its prognosis is the incoherence between macro- and microcirculation, which occurs when microcirculatory abnormalities persist after macrohemodynamic parameters have returned to normal, leading to tissue hypoperfusion and impaired oxygen delivery to cells.^(5,6)^ Several events related to microcirculatory failure have been proposed, such as changes in vascular reactivity, endothelial dysfunction, glycocalyx barrier degradation, and microthrombosis.^(6-8)^ However, evidence of these mechanisms in human studies are rare in the literature.^(9-11)^

When there is a loss of hemodynamic coherence, lactate can be used to assess systemic organ perfusion,^(12,13)^ but hyperlactatemia may also be explained by other factors not related to a decrease in oxygen delivery.^(7)^ Therefore, peripheral perfusion can be easily monitored at the bedside using capillary refill time (CRT)^(14,15)^ or the peripheral perfusion index (PI).^(16,17)^ Prior research have demonstrated a strong association between CRT and PI and outcome in sepsis.^(14,18)^ Furthermore in the presence of persistent hyperlactatemia,^(13)^ CRT indicates the subgroup with the highest mortality,^(15)^ as does microvascular reactivity assessed by PI.^(19)^

Additionally, the role of mediators that interfere with immune activity has been discussed, particularly regarding the pathways leading to dysregulated response to infection.^(3,4,20)^ Some mediators, known as damage-associated molecular patterns (DAMPs or alarmins), are host endogenous molecules released by the immune cells due to necrosis, tissue damage or cellular stress secondary to the inflammatory response.^(21,22)^ In order to activate the innate immune system and eliminate the aggressor, DAMPs bind to cell receptors, triggering a complex signaling cascade that may also lead to immune and inflammatory dysfunction. These processes are frequently linked to multiple organ dysfunction and can worsen prognosis.^(3,21,22)^

Therefore, considering that a) persistent microcirculatory alterations are associated with high mortality in sepsis, particularly when macro- and microcirculation become uncoupled; b) the underlying mechanisms of these alterations are not well evidenced in human studies, and c) DAMPs are associated with immune dysregulation and may theoretically be associated with microcirculatory dysfunction in sepsis, this study aimed to verify the potential association between serum levels of DAMPs and hypoperfusion in septic patients.

## OBJECTIVE

The purpose of this study was to determine whether HMGB1, histone H3, and histone H4 are associated with peripheral hypoperfusion in septic patients.

## METHODS

A multicenter retrospective cohort study was conducted using a database and stored blood samples from patients enrolled in a previous major study protocol,^(19)^ which is registered in the Brazilian Registry of Clinical Trials under the code RBR-35tv9ft. The study was approved by the Human Research Ethics Committee of the *Hospital de Clínicas* of the *Universidade Federal do Paraná*, (CAAE: 28824120.3.0000.0096; #3.913.982). This manuscript also adheres to The Strengthening the Reporting of Observational Studies in Epidemiology (STROBE) Statement.

The study included patients aged 18 years or older who were admitted to three intensive care units at a public tertiary hospital and at a private hospital in southern Brazil, between 2021 and 2022. Patients had sepsis or septic shock within the first 24 hours of diagnosis and received initial hemodynamic resuscitation in accordance with international guidelines.^(1,20)^

The exclusion criteria were defined at the implementation of the previous study protocol and included severe cirrhosis (Child Pugh class C); severe coagulopathy (platelets <20.000/mm3, activated partial thromboplastin time >70 seconds or International Normalized Ratio >2); severe active bleeding (such as hemorrhagic stroke and upper gastrointestinal bleeding); endocarditis; severe peripheral arterial insufficiency, scleroderma and pregnancy.

All patients or their legal surrogates provided written informed consent.

Epidemiological, clinical, and laboratory data were collected throughout hospitalization. Blood samples for the assessment of serum DAMPs and peripheral perfusion measurements were obtained simultaneously at a single time point: within the first 24 hours after a diagnosis of sepsis or septic shock and after hemodynamic stabilization and adequate fluid resuscitation.

Plasma measurements of three DAMPs - histone H3, histone H4 and high-mobility group box-1 protein (HMGB1) - were performed on the available samples using specific enzyme-linked immunosorbent assay (ELISA) kits according to the manufacturer's instructions: histone H3 (Cayman Chemical, Ann Arbor, MI, USA), histone H4 (EpigenTEK, Farmingdale, NY, USA) and HMGB1 protein (Elabscience, Houston, TX, USA).

Peripheral hypoperfusion was evaluated using PI and CRT values. A pulse oximeter (Masimo Radical®, Masimo-Corp, USA, and Mindray, China) was used to measure PI. The cut-off value defined as hypoperfusion was less than 1.4 for PI^(18)^ and greater than 3 seconds for CRT.^(14)^

The association between HMGB1, histone H3, histone H4, and peripheral perfusion markers (CRT and PI) was the primary outcome. The correlation between DAMPs and mortality as well as the relation between peripheral perfusion variables and mortality were defined as secondary outcomes. We also examined the patients into shock and non-shock subgroups in a *post-hoc* analysis to search for an association between peripheral perfusion and DAMPs.

Sample size calculation was not feasible because the study was based on the database of a previous study protocol. Statistical analyses were performed using GraphPad Prism 10 software. Means and standard deviations were used to characterize quantitative variables, whereas absolute and relative frequencies were used to characterize categorical variables. The Shapiro-Wilk test was used to evaluate the normality of the variables. Since all the independent variables did not have a normal distribution, non-parametric data were described as median and interquartile range, and the Mann-Whitney U-test was the statistical test applied for all the analyses, with a 95% confidence interval and a 5% threshold of statistical significance.

## RESULTS

Eighty patients were included in the study, with a mean age of 61 years, and half of them were male. Most patients had some type of comorbidity. The clinical variables are summarized in table 1.

**Table 1:** Baseline characteristics of the patients

Epidemiological and clinical characteristics		n=80
Female, n (%)		40 (50)
Age, mean±SD		61±16.5
Comorbidities, n (%)		64 (80)
	Hypertension	44 (55)
	*Diabetes mellitus*	28 (35)
	Heart failure	10 (12.5)
	Chronic obstructive pulmonary disease	7 (8.75)
Source of infection, n (%)		
	Abdominal	28 (35)
	Pulmonary	23 (28.75)
	Urinary	16 (20)
	Others	13 (16.25)
Severity scores, mean±SD		
	SOFA score	7±3
	APACHE II score	18±6
Hemodynamic variables		
	Mean arterial pressure, median (IQR), mmHg	81 (73-93)
	Heart rate, mean±SD, bpm	97±21
	Urine output, median (IQR), ml/kg/h*	0.41 (0.20-0.79)
Vasoactive drugs use, n (%)		39 (48.75)
Norepinephrine dosage, median (IQR), μg/kg/min**		0.25 (0.13-0.60)
Septic shock, n (%)		25 (31.25)
Mortality, n (%)		30 (37.5)

* Missing Data n=76;

** Missing Data n=39.

SD: standard deviation, SOFA: Sequential Organ Failure Assessment, APACHE II: Acute Physiology and Chronic Health Evaluation.

When perfusion parameters were assessed, more than half of the patients had persistent hyperlactatemia despite adequate fluid resuscitation, 45% had prolonged CRT, and approximately 50% had hypoperfusion as determined by PI (Table 2). Perfusion index <1.4, extended CRT, and hyperlactatemia were all indicators of hypoperfusion in 25% of the patients.

**Table 2:** Perfusion data

Persistent hyperlactatemia, n (%)		
	Yes	38 (47.5)
	No	42 (52.5)
CRT >3 s, n (%)		
	Yes	36 (45)
	No	44 (55)
PI <1.4, n (%)		
	Yes	38 (49.35)*
	No	39 (50.65)*

* Missing data: total n=77.

Variables assessed after hemodynamic stabilization and adequate fluid resuscitation. Persistent hyperlactatemia was considered as a lactate level above 2mmol/L. Results in n (%).

**Figure 1:**
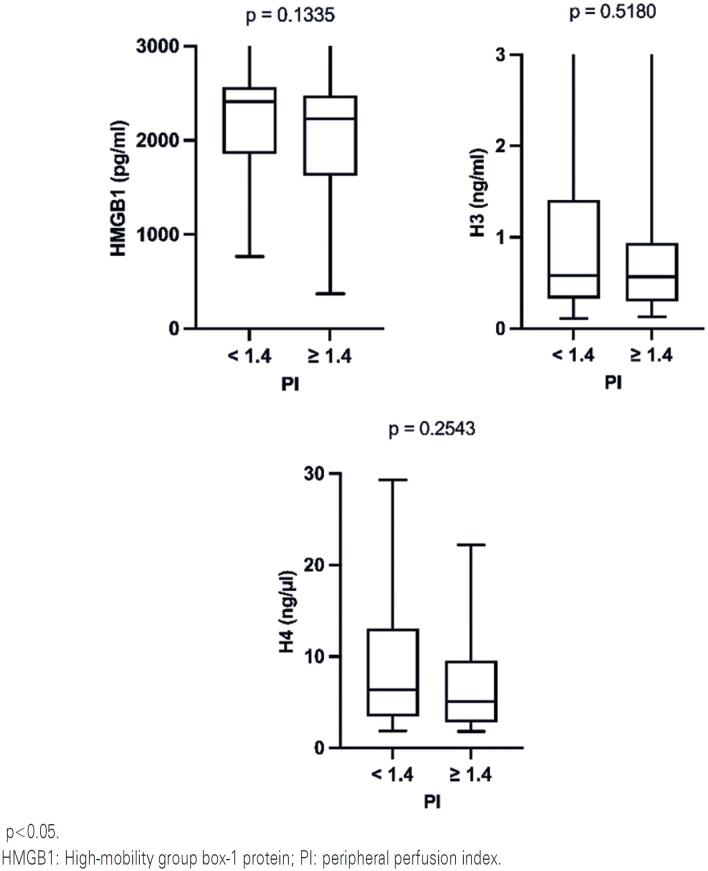
Association between HMGB1, histone H3 and histone H4 with peripheral perfusion index

**Figure 2:**
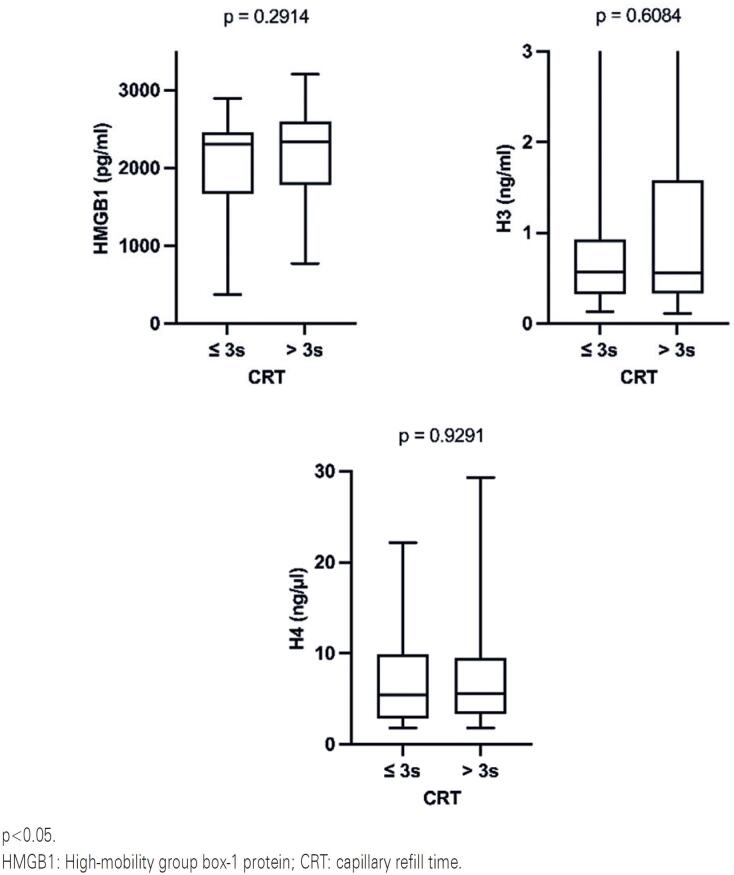
Association between HMGB1, histone H3 and histone H4 with capillary refill time

There was a lack of statistical association between the evaluated DAMPs and PI (Figure 1) or CRT (Figure 2). Additionally, when the patients were separated into shock and non-shock subgroups, the results indicated no difference between DAMPs and either PI or CRT in these subgroups (p>0.05 in all data analyzed).

Moreover, there were no statistically significant associations between mortality and serum levels of histones H3, H4, or HMGB1 (Table 3). Nonetheless, there was a statistically significant relation between all the perfusion variables and mortality, as indicated in table 4.

**Table 3:** Association between HMGB1, histone H3 and histone H4 with mortality

	Survivors (n=50)	Non-survivors (n=30)	p value
HMGB1, pg/ml	2185 (1570-2530)	2377 (2124-2508)	0.08
Histone H3, ng/ml	0.58 (0.38-1.07)	0.54 (0.29-0.91)	0.44
Histone H4, ng/µl	4.67 (2.71-9.54)	6.88 (3.77-11.97)	0.08

p<0.05.

Results expressed as median and interquartile range. HMGB1: High-mobility group box-1 protein.

**Table 4:** Association between perfusion data and mortality

	Survivors (n=50)	Non-survivors (n=30)	p value
CRT, s	2 (2 - 4)	4 (2 - 5)	0.0124
PI*	1.90 (0.98-4.95)	0.54 (0.32-0.87)	<0.0001
Lactate, mmol/l	1.55 (1.23-2.52)	2.89 (1.69-4.44)	0.0009

p<0.05.

* Missing data: n survivors=49; n non-survivors=28.

Results expressed as median and interquartile range.

CRT: capillary refill time; PI: peripheral perfusion index.

## DISCUSSION

Persistent tissue hypoperfusion is clearly related to increased mortality^(23)^ and its mechanisms are still unclear. On the other hand, DAMPs are currently recognized as important factors in the immune-inflammatory response in sepsis and are closely associated with tissue damage and multiple organ dysfunction.^(4,22)^ In addition, previous experimental studies have demonstrated that modulating DAMPs could improve the prognosis in sepsis.^(24,25)^ Therefore, this study was the first to assess whether serum levels of DAMPs are related to hypoperfusion during the hemodynamic uncoupling phase in septic patients. Nevertheless, we found no association between tissue hypoperfusion and plasma levels of HMGB1, histone H3, and histone H4.

During sepsis, organ hypoperfusion is a dynamic event. In the early phase of resuscitation, hypoperfusion is more dependent on macrohemodynamics (flow-dependent), whereas local metabolic variables predominate in the later stages.^(7-9,19)^ To highlight the microcirculatory mechanisms, which can be observed by the stability of the sample's macrohemodynamic parameters after fluid resuscitation — our study focused on evaluating this later phase. Additionally, peripheral perfusion is the first to be impaired and the last to recover due to the redistribution of blood flow in sepsis,^(18,19)^ so, peripheral hypoperfusion could be a marker of this redistribution. Although our study did not directly measure the cardiac output, the stability of the macrohemodynamic variables makes this explanation less likely for the results.

Several mechanisms have been described to explain these persistent microcirculatory alterations in sepsis,^(7-11,23)^ leading to a heterogeneity of microvascular blood flow, endothelial cell injury, and impaired vascular reactivity to local vasoconstriction-dilation mediators.^(9)^ Previous studies have shown that the excessive release of histones and HMGB1 can damage the structure and function of the endothelium,^(11,25)^ and, at least theoretically, DAMPs could be a linking factor between innate immune activation and the development of microcirculatory failure. However, the lack of difference in DAMPs levels between patients with reduced and normal peripheral perfusion may suggest that DAMPs release could be the consequence rather than a cause of microvascular alterations, representing an "epiphenomenon", since microcirculation failure is an independent predictor of outcome, well described in the literature.^(5,6,15,18,19)^ Additional studies are needed to confirm these conclusions.

These assumptions may also be supported by the fact that an increasing amount of evidence correlates DAMPs with critical illnesses directly related to cell death triggered by the inflammatory process, such as different types of necrosis (pyroptosis, necroptosis and ferroptosis), rather than directly with tissue hypoperfusion.^(26-28)^ Thus, DAMPs could act as a marker of the initial innate immune-inflammatory insult that contributes to a cascade of organ damages, the latter responsible for mortality.^(22)^ This hypothesis is reinforced by evidence regarding another biomarker associated with the innate immune response, C-reactive protein, which is related to the development of sepsis but not to its prognosis.^(29)^

Another assumption deserves exploration. Since the proportion of perfused capillaries, a strong predictor of sepsis prognosis, cannot distinguish between survivors and nonsurvivors during the first 24 hours of septic shock, even though the value that differentiates these patients rises with time,^(5,6)^ there is another hypothesis associated with DAMPs measurement at a single time point. The DAMPs examined in our investigation were unable to distinguish between septic patients with hypoperfusion and those with normoperfusion, which may be explained by the fact that HMGB1, histone H3, and histone H4 serum levels were tested within the first 24 hours of illness. The association between HMGB1 and sepsis prognosis^(30-34)^ is another finding that supports this theory. In contrast to early measurement, studies that analyzed HMGB1 serially consistently indicated that late HMGB1 measurement was associated with mortality, unlike the initial measurement.^(31,35,36)^

Regarding the histones H3 and H4, the results also contrasted with the literature.^(33,34)^ However, a recent review demonstrated that it is not possible to clearly confirm this association, given the lack of a gold standard technique and the variety of methodologies used to quantify histones in studies^(37)^ Furthermore, efforts to reduce publication bias have attracted interest in the literature, as it affects the magnitude of evidence, reducing the precision of clinical management.^(38)^ Thus, our negative findings may contribute to a subsequent meta-analysis focusing on the real prognostic accuracy of histones.

In addition, the concept of "enrichment" in precision medicine regarding sepsis is another interesting aspect related to our results.^(39)^ Predictive enrichment prioritizes subgroups based on the same pathophysiological process (immunosuppression, for example), whereas prognostic enrichment focuses on a higher likelihood of a hard endpoint (such as mortality).^(39)^ Therefore, our findings suggest that enrichment for any treatment guided by DAMPs measurements in sepsis should be predictive rather than prognostic, as it occurs with perfusion parameters.

Our study has some important limitations. First, we were unable to determine the sample size necessary to ensure the statistical power of the study because we had limited resources for processing serum DAMPs, although our findings suggest that the effect size would likely have minimal clinical significance if an association were found in a larger group. Second, the fact that a single serum measurement of DAMPs was performed during the early stage of sepsis does not exclude the possibility that DAMPs and peripheral hypoperfusion could be associated in an eventual serial analysis, as discussed above. Third, although the dose of noradrenaline in the study was similar in both groups, the use of noradrenaline as a vasopressor may affect the microcirculation, causing a stopped flow in capillaries as a result of excessive vasoconstriction, directly impairing tissue perfusion.^(6)^ Additionally, only one-third of patients met the criteria for septic shock after initial hemodynamic stabilization,^(20)^ which may indicate a subgroup with a poor prognosis. However, our statistical analysis suggests that this was independent of DAMPs levels. Finally, the patients included in our study were from hospitals in a middle-income country, which is known to affect sepsis prognosis.^(40)^ Consequently, it is not feasible to generalize these findings to countries with more or fewer resources.

## CONCLUSION

In conclusion, our findings demonstrated that among septic patients, there was no statistically significant association between the levels of histone H3, histone H4, and HMGB1 protein and hypoperfusion or mortality.

## Data Availability

Data are available to reviewers upon request.
